# Impact of COVID-19 restrictions on health and well-being in the United Arab Emirates

**DOI:** 10.3389/fpsyg.2023.1259974

**Published:** 2023-11-03

**Authors:** Sarah Dalibalta, Nariman Ghader, Layal Rabah, Sami Shaban, Noor Al Mheiri

**Affiliations:** ^1^College of Arts and Sciences, American University of Sharjah, University City, Sharjah, United Arab Emirates; ^2^Department of Mental Health, Medical Services Sector, Emirates Health Services, Dubai, United Arab Emirates; ^3^College of Medicine and Health Sciences, United Arab Emirates University, Al Ain, United Arab Emirates

**Keywords:** COVID-19, social distancing, physical health, well-being, United Arab Emirates

## Abstract

**Background:**

Restrictions during the COVID-19 pandemic are thought to have impacted both the physical health and well-being of individuals where lockdown was applied. The United Arab Emirates (UAE) was one of the leading countries in implementing the international guidelines to limit the intensely contagious nature of the outbreak.

**Aim:**

To identify the impact of COVID-19 on changes to exercise and general physical activity habits, changes to the consumption of various foods and potential weight gain, as well as any differences in smoking habits among individuals residing in the UAE during the COVID-19 quarantine.

**Methods:**

This is a cross-sectional analytical study that used a quantitative electronic questionnaire sent by the Ministry of Health and Prevention to individuals on its platform in order to collect data on the physical health and well-being of a UAE sample population. A total of 2,362 responses were received to specific questions on physical activity, eating habits, and tobacco use for the period before, during, and after the COVID-19 lockdown. Descriptive statistical analysis was used to display the sample’s demographic data and the changes in physical health and well-being. Paired *t*-test was used to show the changes in dietary habits.

**Results:**

This study reveals concerning changes in health risk behaviors during the COVID-19 lockdown in the UAE. Physical activity levels declined across mild, moderate and vigorous ranges in most participants. Alarmingly, sedentary behavior dramatically increased with 71% of participants spending an average of 4–8 h per day sitting and over 54% of participants spending more than 4 h watching TV on an average day during lockdown. Fast-food consumption and snacking rose, hence weight gain was observed in over 53% of participants. Smoking habits, especially among cigarette smokers, may have worsened, with 45.2% reporting an increase in cigarette smoking, 16.8% declaring an increase in shisha smoking and 35.3% reporting an increase in smoking other tobacco products. These unfavorable behaviors during confinement could have serious long-term health consequences.

**Conclusion:**

This study demonstrates that long periods of home quarantine may have led to unhealthy consequences that increase the risk of developing disease. This study therefore aims to highlight these health impacts, and recommend strategies and policies that can encourage healthy habits.

## 1. Introduction

In March 2020, the coronavirus (COVID-19) outbreak that began in China in December 2019 started to force countries into strict lockdown measures. This resulted in universal impacts on social activity, physical activity (PA), dietary habits as well as mental health (Pellegrini et al., 2020; Romero-Blanco et al., 2020; Radwan et al., 2021).

The United Arab Emirates (UAE) responded to the pandemic by initiating a general lockdown by mid-March 2020, implementing social distancing, distance learning, and sanitization programs like other countries worldwide (Cheikh Ismail et al., 2020). The confinement period significantly restricted outdoor physical activity and promoted sedentary habits. Even prior to the COVID-19 pandemic, a quarter of the UAE population was reported to have a sedentary lifestyle and were not engaged in any type of PA (Yammine, 2017). Therefore, the potential consequences of further movement restrictions could be detrimental to this population. Moreover, spending time indoors contributed to boredom and exacerbated overeating (Bakhsh et al., 2021). Due to quarantine measures, the nutritional status of populations globally had been affected leading to deficiencies in the intake of micro and macronutrients (Pellegrini et al., 2020; Bakhsh et al., 2021; Wilke et al., 2021). An international online survey in April 2020 sent to thirty-five research organizations from Europe, North-Africa, Western Asia and the Americas in eight languages concluded negative effects on physical activity and increased daily sitting times (Ammar et al., 2020). Moreover, the consumption of unhealthy foods and snacking between meals was shown to be exacerbated. These reports were alarming despite recommendations from the World Health Organization (WHO) and significant technologies (apps, social media, online communication channels) disseminating large quantities of information. For instance, a large number of videos on YouTube showed that home-based exercises were a safe way to perform physical exercise during lockdown (Vancini et al., 2021). The authors recommended however that although online sources may be a powerful tool for propagating knowledge and health suggestions, information should be accurate and from reputable sources and should be aligned with scientific sources In the UAE, 41% of surveyed COVID-19 patients were categorized as overweight and 25% were obese. Consequently, these overweight/obese patients had a 58% higher risk of developing severe COVID-19 symptoms compared to normal-weight patients (Al Bastaki et al., 2022). Hence, although lockdown protocols aimed to safeguard populations from COVID-19 risk, the low levels of physical activity and social isolation also worsened respiratory and cardiovascular health risk factors; as well as psychological stress, anxiety, and depression (Abouzid et al., 2021; López-Bueno et al., 2021; Stockwell et al., 2021; Al Sabbah et al., 2022). These changes resulted in a rise in smoking rates as a coping mechanism among users and non-users, although smokers had a higher chance of developing COVID symptoms due to constant lung infections and lower immunity (Al Hazzaa, 2004; Al Sabbah et al., 2022). Generally, lifestyle changes like dietary habits, smoking habits, and reduced levels of physical activity can also affect the immune system and increase the risks of obesity and coronary heart disease (Damiot et al., 2020; Okati-Aliabad et al., 2022; Schäfer et al., 2022). These effects were also observed in athletes whereby both elite and amateur athletes had lower levels of inactivity during the pandemic with noted changes to their dietary habits, sleep quality and stress levels (Taheri et al., 2023).

To investigate the implications of the COVID-19 lockdown on the health and well-being of the UAE population, this study aimed to identify the impact of COVID-19 on physical activity levels, eating habits, weight gain, and smoking habits among individuals residing in the UAE during the COVID-19 quarantine. A comparison of lifestyle and dietary behaviors before, during, and after the lockdown period was also conducted to allow for a better understanding of the effects of COVID-19-induced confinement policies, which may aid in setting suitable recommendations for this population during confinement periods in the future. In fact, research funding and governmental priorities have been adjusted worldwide to better prepare for health emergencies (Weiner et al., 2020). As such, areas of high priority have been established to enhance biomedical research, establish economic resilience, create innovative strategies and technologies to revolutionize healthcare systems and build a research infrastructure that can better deal with disasters and public health emergencies.

## 2. Materials and methods

In this cross-sectional analytical study, a quantitative electronic questionnaire was developed to measure the impact of COVID-19 restrictions on health and well-being in the UAE. The tool consisted of several sections comprising general demographics and health-related information (weight and physical activity, eating habits, and tobacco use) for the period before, during, and after the COVID-19 lockdown. The full questionnaire is available upon request from the authors with key questions stated in Tables 1–5.

**Table 1:** Demographic variables of study participants.

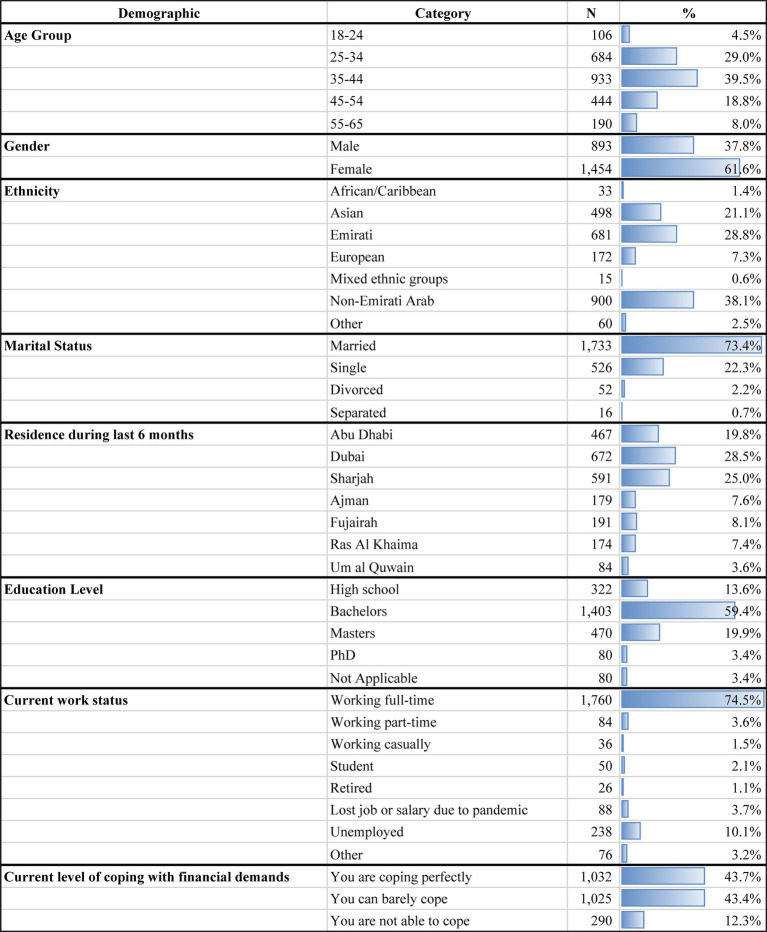

**Table 2:** Physical activity and sedentary behavior changes of participants.

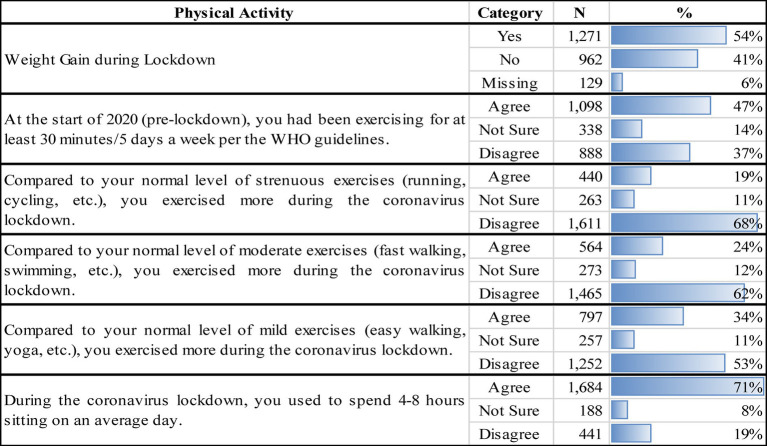

**Table 3:** Changes in dietary habits using paired *t*-test.

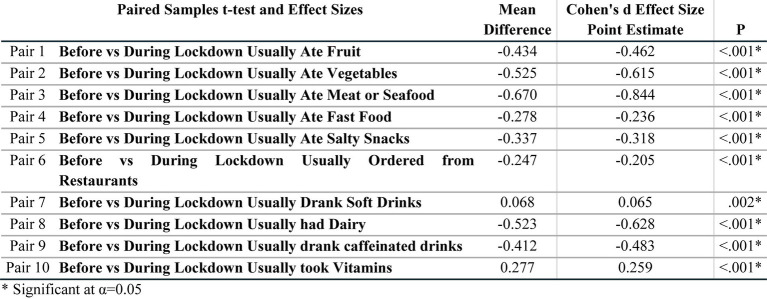

**Table 4:** Changes in smoking habits of participants.

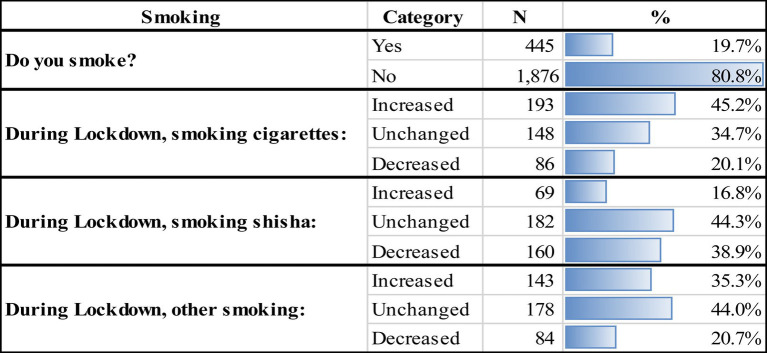

**Table 5:** Changes in physical activity, dietary habits, and smoking post lockdown (during social distancing period).

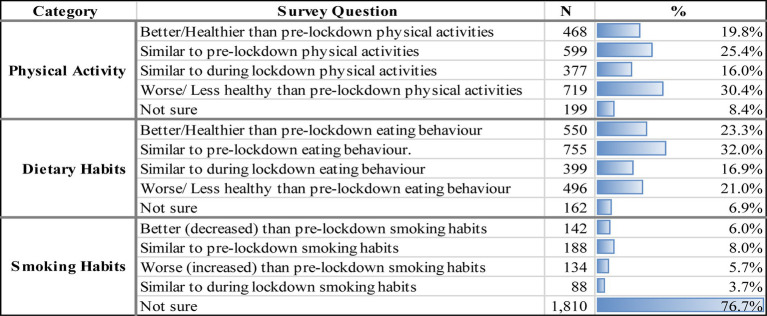

The call to participate in the study, which extended from October 2020 to April 2021, included a participant information sheet, an informed consent, and a questionnaire, which should take no more than 30 min to complete. Invites were sent out to the UAE population *via* the electronic communication means of the UAE Ministry of Health and Prevention and the American University of Sharjah. Individuals who met the inclusion and exclusion criteria as informed in the participant information sheet and ticked the “Accept” box in the informed consent were included in the data analysis. A total number of 2,400 responses (self-selection) were obtained *via* the electronic data collection platform, however, only 2,362 entries were analyzed after data cleaning using IBM SPSS Statistical Software. Descriptive statistics with frequencies and percentages were calculated for all variables (Tables 1–5). A paired *t*-test (*p* ≤ 0.05) was used to compare changes in eating habits before and during lockdown (Table 3).

## 3. Results

Data from a population sample of 2,362 participants were included in this study. Questions about the participants’ age, gender, ethnicity, financial and marital status, education, and work were analyzed. Further analysis also included the participants’ physical activity, diet, and smoking habits.

### 3.1. Participants’ information

The number of respondents and percentages may vary slightly among the sections due to missing responses from some participants. As shown in Table 1, most of the participants were between the ages of 35–44 years old with 61.6% (*n* = 1,454) being females and 37.8% being males (*n* = 893). The ethnicities of the participants varied, with 38.1% non-Emirati Arabs (*n* = 900), 28.8% Emiratis (*n* = 681), 21.1% Asians (*n* = 498), 1.4% African/Caribbean (*n* = 33), 7.3% European (*n* = 172), 0.6% from mixed ethnic groups (*n* = 16) and 2.5% identified as other (*n* = 60). Generally, participants were married (*n* = 1,733, 73.4%) and resided mostly in Dubai (*n* = 672, 28.5%), Abu Dhabi (*n* = 467, 19.8%), and Sharjah (*n* = 591, 25.0%). The majority had at least a bachelor’s degree (*n* = 1,403, 59.4%), 19.9% had a master’s degree (*n* = 470), and 3.4% had a Ph.D (*n* = 80). Most were employed and working full time (*n* = 1,760, 74.5%), with 10.1% unemployed at the time of the survey and 3.7% claiming to have lost their job due to the pandemic. Moreover, around 56% of surveyed participants reported barely coping with financial demands during that period, which in doubt leads to both mental and physical stress.

### 3.2. Changes in physical activity habits

The survey was used to assess changes in the levels of physical activity and sedentary behavior before and during the COVID-19 lockdown period. Physical activity can be defined as any form of movement that involves energy expenditure caused by skeletal muscles including walking, commuting to work/schools, cycling, and playing sports (World Health Organization, 2022). It also classifies PA according to intensity into the light, moderate, and vigorous ranges. According to the WHO guidelines, an adult should aim for an average of 150–300 min of moderate-intensity aerobic physical activity (PA) and/or 75–150 min of vigorous/strenuous-intensity aerobic PA per week (World Health Organization, 2022). Adults should also incorporate muscle strength training focusing on all major muscle groups for two or more days weekly to maintain a healthy lifestyle and decrease the risk of infections and mortality (World Health Organization, 2022).

Table 2 describes changes in physical activity before and during lockdown among participants. Results reveal that before the pandemic, 46.5% of participants had been engaging in PA for at least 30 min/5 days a week, hence meeting the WHO guidelines, in contrast to 36.6% who reported not meeting the minimum guidelines for PA. During the lockdown, 33.7% stated that they did more PA in the mild range (such as easy walking, yoga etc.) compared to pre-lockdown. Moreover, 23.9% did more moderate-intensity PA (fast walking, swimming, etc.) and 18.6% did more strenuous PA (high-intensity exercises like running, cycling, etc.) during lockdown compared to the pre-lockdown period. Therefore, the majority of participants reported to have exercised less in the mild (53.1%), moderate (62%), and strenuous (68.2%) ranges compared to normal levels pre-lockdown.

Sedentary behaviors can be defined as a distinct class of behaviors characterized by low energy expenditure and include activities such as sleeping, sitting, lying down, and watching TV (Pate et al., 2008). Results revealed that 71.3% of participants spent an average of 4–8 h per day sitting during lockdown with the majority agreeing that they spent significantly more time sitting during lockdown compared to pre-lockdown. Data also demonstrated that 54.5% of participants spent more than 4 h watching TV on an average day during lockdown. It is therefore evident that participants were extremely sedentary during the lockdown period, and this has been correlated with adverse health outcomes and increased risk of cardiovascular morbidity and mortality (Pate et al., 2008). Hence, it is unsurprising that 53.8% of surveyed participants reported an increase in weight during lockdown.

### 3.3. Changes in dietary habits

The survey also aimed to observe changes in dietary habits before and during the COVID-19 lockdown by exploring questions on the intake of fruits, vegetables, meat/seafood, fast food, restaurant food, salty snacks, soft drinks, caffeinated drinks, dairy products, and vitamin supplements. Table 3 shows descriptive statistics representing the *p*-values of paired *t*-tests for changes in dietary habits pre and during lockdown. Results reveal statistical differences in all of these eating behaviors including vitamin intake. Data shows a statistical difference in the intake of fruits and vegetables before and during lockdown with fruit and vegetable consumption increasing during the lockdown period.

Likewise, the intake of meat, seafood, and dairy during lockdown was statistically higher compared to pre-lockdown. The consumption of fast food and salty snacks also significantly increased during the lockdown period. Furthermore, participants were more likely to order more from restaurants throughout confinement compared to pre-lockdown. On the other hand, the intake of soft drinks was statistically lower during the lockdown, although participants seemed to increase their consumption of caffeinated drinks compared to the pre-lockdown period.

Overall, some aspects of diet appeared to have improved during the lockdown period as shown in the participants’ increase in the consumption of fruits, vegetables, meat, and seafood and decrease in the intake of soft drinks. This may be attributed to individuals becoming more health conscious as well as spending more time at home cooking. However, it is also noteworthy that fast food, restaurant delivery, and snacking were shown to be higher during lockdown possibly contributing to the weight gain reported in half of the surveyed participants.

### 3.4. Changes in smoking habits

In Table 4, smoking habits were evaluated through questions on the smoking of cigarettes, shisha, and other types of tobacco products before and during lockdown. Results demonstrate that only 19.7% (*n* = 445) of participants were smokers and 80.83% (*n* = 1,876) were non-smokers. During the lockdown, 45.2% of smokers reported an increase in cigarette smoking compared to 20.1% who reported a decrease in their smoking habits. As for smoking shisha, 16.8% of surveyed participants declared an increase in their intake of shisha during lockdown whereas 44.3 and 38.9% reported no change or a decrease in their shisha habits, respectively. As for smoking other tobacco products, 35.3% reported an increase in their smoking habits, 44% remained unchanged, and 20.7% reported a decrease in their smoking habits during lockdown. Overall, although a relatively small percentage of participants were smokers, the majority surprisingly did not decrease their intake of cigarettes, shisha, or other tobacco products even when faced with a virus that primarily affects the respiratory system.

### 3.5. Changes in physical activity, diet, and smoking habits post lockdown (social distancing phase)

In addition to questions about habit changes during the lockdown, the survey also asked participants about how their physical activity levels, dietary habits, and smoking habits may have changed once the lockdown was lifted and a social distancing period resumed. Results reveal that 30.4% of participants felt that their physical activity had worsened compared to pre-lockdown. On the other hand, 25.4% mentioned that their physical activity was somewhat similar to the pre-lockdown period and 16% felt it was like PA during the lockdown period. About 19.8% reported healthier physical activity levels compared to pre-lockdown. In their diets, only 23.3% reported a healthier diet post-lockdown, with 16.9 and 32% mentioning that their eating habits were similar to pre-lockdown or during lockdown, respectively. Interestingly, 21% stated their diets had worsened post-lockdown. Lastly, among smokers, 5.7% mentioned that their smoking habits worsened during the social distancing phase, with only 6% reporting a decrease in smoking habits post-lockdown. However, 8 and 3.7% stated that their smoking habits were similar to pre-lockdown or during lockdown, respectively (Table 5).

## 4. Discussion

At the end of 2019, coronavirus SARS-CoV-2 (COVID-19) spread throughout the world forcing a worldwide lockdown that affected physical and mental health globally (Young et al., 2016; Abouzid et al., 2021; Laddu et al., 2023). This study shows demonstrable changes in physical activity, sedentary behavior, diet, and smoking habits within the UAE. Data revealed that although almost half of the surveyed participants were active and meeting the WHO guidelines of 150–300 min 5 days/week pre-lockdown, the majority were quite inactive during the lockdown and did not recover to pre-lockdown status after the restrictions were lifted. In fact, most of the participants surveyed were particularly sedentary, spending long periods sitting or watching TV. Similarly, a survey conducted among 10 Arab countries (Bahrain, Egypt, Jordan, Kuwait, Lebanon, Oman, Palestine, Qatar, Saudi Arabia, and the United Arab Emirates), stated that within the UAE moderate activity levels decreased from 37.9% pre-lockdown to 34.3% during lockdown and high activity levels decreased from 15.8% pre-lockdown to 9.9% during lockdown (Al Sabbah et al., 2022).

This study also reported that among the 10 Arab countries surveyed, there was an overall decline in physical activity levels and an increase in the percentage of physically inactive participants within all countries during lockdown. Similarly, a survey study in the MENA region showed that 48% of participants did not take part in physical activity prior to the onset of COVID and that there was a 42% increase in the number of participants that seized practicing sports during lockdown (Abouzid et al., 2021). Additionally, a study in Saudi Arabia reported that the percentage of active individuals declined from 54.3 to 47.3% during confinement (Hamed et al., 2022). Global data also showed similar trends with the UK reporting that 45% of the surveyed participants increased their exercising habits during lockdown whereas 40% decreased their activity level (Laddu et al., 2023).

Physical inactivity can have a serious impact on health especially on cardiovascular health and may result in long-term health risks such as a 24% increased risk of coronary heart disease, a 16% increased risk of stroke, and a 42% increased risk of diabetes (Lippi et al., 2020; Robinson et al., 2021). Sedentary behavior is also a major health risk and can lead to a rise in mortality globally (Woods et al., 2020). These results show an alarming increase in sedentary behavior, with over half of participants reporting sitting time of 4–8 h daily confinement and a rise in screen time of over 4 h daily. In Spain, a survey during lockdown also reported that participants’ daily screen time exceeded recommended levels for adults, which may contribute to mental health disorders (López-Bueno et al., 2021).

Weight gain was also reported in about 53.8% of respondents, which may accompany the lower PA levels as well as changes to eating habits during the lockdown. Results show a rise in consumption of fruits, vegetables, meat, seafood, and dairy and in snacking, fast food, and restaurant takeaways. About 21% of participants also report dietary habits declining post-lockdown, during the social distancing phase, with only 23.3% adjusting to a healthier diet after lockdown. Similarly, a study in Saudi Arabia reported that 40% of the 2,255 adults surveyed had increased their consumption of food and 45% snacked more during lockdown resulting in weight gain (Bakhsh et al., 2021). 73% of the adults surveyed stated that they relied on home-cooked meals, of which 47% attained healthy meals and 7% declined their intake of restaurant food during lockdown (Bakhsh et al., 2021). Equally, a survey in the MENA region stated that 41.6% of participants consumed more fruits and vegetables during the lockdown (Abouzid et al., 2021) and a survey in the UAE indicated that 2/3 of the surveyed participants increased their consumption of citrus fruits during COVID (Radwan et al., 2022).

There was also a statistically significant change in vitamin supplementation pre or during confinement which is in line with other surveys conducted in the Middle East involving 5 countries (Lebanon, Saudi Arabia, Palestine, Jordon, and the United Arab Emirates). They reported that 77.8% of surveyed adults were taking vitamin C supplements and about 46.6% believing that dietary supplements were useful in preventing COVID (Mukattash et al., 2022). Radwan et al. (2022), in a UAE survey, reported that 56.6% of the 2,060 participants confirmed the use of dietary supplements: vitamin C, vitamin D, and multivitamins as a form of prevention or treatment during COVID-19 (Radwan et al., 2022).

Data also showed that most of the smokers surveyed cited an increase in their smoking habits during confinement, particularly in cigarette smoking. Moreover, about a third of smokers stated an increase in other types of tobacco smoking while being on lockdown. Post-lockdown, there was a slight improvement in the smoking habits of 6% of participants, although others reported that they either increased their smoke intake or maintained it like pre and during-lockdown periods. Similarly, a survey in the US reported that among cigarette smokers, 33% stated an increase in their habits due to increased stress caused by the pandemic, with 23% reporting an increase in e-cigarette use (Kalkhoran et al., 2022). On the contrary, in 10 Arab countries in the Middle East, the average smoking rate decreased by 24% during lockdown (Al Sabbah et al., 2022).

## 5. Conclusion

In conclusion, this study reports significant changes during the COVID-19 lockdown that may have profound consequences to the UAE population in the future. A set of health risk behaviors were studied and showed that these increased during confinement. Recommended physical activity levels declined whilst sedentary behavior and screen time significantly increased. Fast food, restaurant takeaway, and snacking had also increased, and so did weight gain, in more than half of participants. Moreover, smoking habits seemed to have boosted among smokers, especially cigarette smokers, and may not have improved post the confinement period. The authors strongly advocate for the formulation of comprehensive strategies that not only provide knowledge and techniques but also assist individuals in adapting and improvising during times of crisis, especially when access to regular and public resources (due to lockdowns or curfews) is limited or uncertain. For instance, when outdoor activities are restricted, individuals should be prepared to craft workout routines with minimal or no equipment, create nutritious meals from available pantry items, explore food substitutions, and seek support for smoking cessation. Furthermore, this study emphasizes the need for future research projects to dig deeper into understanding the critical relationship between altered healthy habits and specific factors; such as educational parameters, economic and employment conditions, and medical status. Moreover, vulnerable groups (such as individuals who are mentally challenged, children and adolescents, and women subject to violence and abuse) should be a top priority for further exploration concerning altered healthy behaviors in the context of one of the greatest conundrums of our time, the COVID-19 pandemic. These proposed studies can unearth how different demographic groups experience and respond to crisis-related shifts in physical activity, dietary habits, and smoking. These expanded perspectives would contribute to the development of scientifically supported interventions that address the unique challenges and needs of various population segments, ensuring that health initiatives are inclusive and effective.

## 6. Study strengths and limitations

This study’s distinctive strength lies in its potential contributions to the scientific community, setting it apart from other studies as the only one in the UAE to adopt a comprehensive approach, simultaneously examining changes in physical activity, dietary habits, and smoking habits simultaneously. This is thought to provide a more holistic understanding of how the pandemic impacted people’s lives. More importantly, the comparative analysis scrutinizes lifestyle changes before, during, and after lockdown, shedding light on how behaviors evolved at various stages of the pandemic. These insights hold significant value for policymakers, offering guidance for long-term planning and interventions aimed at strengthening the country’s resilience against uncertainties and future adversities. However, despite its strengths, this study also has some limitations that should be acknowledged. Firstly, due to the comprehensive datasets utilized, the data analysis primarily focused on specific aspects aligned with the initial study objectives. Nevertheless, there remains potential for conducting additional multiple covariate analyses in subsequent follow-up studies, allowing for a more comprehensive exploration of a range of factors. Additionally, a limitation of this study is the potential for self-selection bias. As participants were included based on their voluntary response to the questionnaire, it is possible that those who chose to participate may possess distinctive characteristics compared to non-respondents. To address this limitation, future studies can consider employing more robust sampling methods that promote enhanced representativeness and minimize the impact of self-selection bias.

## Data availability statement

The original contributions presented in the study are included in the article further inquiries can be directed to the corresponding authors.

## Ethics statement

This study was approved by the Research Ethics Committee of the Ministry of Health and Prevention in the United Arab Emirates. The study was conducted in accordance with the local legislation and institutional requirements. The participants provided their written informed consent to participate in this study.

## Author contributions

SD: Conceptualization, Data curation, Formal analysis, Investigation, Methodology, Project administration, Resources, Validation, Writing – original draft, Writing – review & editing. NG: Conceptualization, Data curation, Formal analysis, Funding acquisition, Investigation, Methodology, Project administration, Resources, Software, Supervision, Validation, Visualization, Writing – original draft, Writing – review & editing. LR: Writing – original draft. SS: Formal analysis, Writing – review & editing, Data curation, Software. NM: Writing – review & editing, Funding acquisition, Project administration, Supervision.
